# ‘One for all, all for one’: A tale from the formative years of Scandinavian exercise physiology

**DOI:** 10.1113/EP091943

**Published:** 2024-06-05

**Authors:** Ronan M. G. Berg

**Affiliations:** ^1^ Centre for Physical Activity Research Copenhagen University Hospital – Rigshospitalet Copenhagen Denmark; ^2^ Department of Clinical Physiology and Nuclear Medicine Copenhagen University Hospital – Rigshospitalet Copenhagen Denmark; ^3^ Department of Biomedical Sciences, Faculty of Health and Medical Sciences University of Copenhagen Copenhagen Denmark; ^4^ Neurovascular Research Laboratory, Faculty of Life Sciences and Education University of South Wales Pontypridd UK

1

In Scandinavia, we have a proud tradition of exercise physiology. The period from 1910 to 1920, during which August Krogh (1874–1949) and Johannes Lindhard (1870–1947) provided measurements on cardiorespiratory function and metabolism during exercise in humans, can certainly be considered its birth, at least from a merely Danish perspective. However, it is in my opinion the decades that followed the establishment of the Rockefeller Institute in Copenhagen in 1928 that comprise the formative years of Scandinavian exercise physiology. This institute, established with funds secured by August Krogh after he became a Nobel laureate, offered advanced research and teaching facilities in areas such as zoophysiology, medical physiology, gymnastics theory, biochemistry and biophysics (Asmussen, 1987). Krogh was instrumental in its design, ensuring the inclusion of facilities for Lindhard's Gymnastics Theory Laboratory on the first floor of the north wing, strategically located above his own Zoophysiological Laboratory to facilitate interaction and collaboration.

It was in this environment, and under the guidance of both Lindhard and Krogh, that three scientists emerged as seminal figures in Scandinavian exercise physiology: Erik Hohwü Christensen (1904–1996), Marius Nielsen (1903–2000) and Erling Asmussen (1907–1991), fondly referred to by Krogh as ‘The Three Musketeers’ (Schmidt‐Nielsen, 1995). The tales of these three scientists are many, and their efforts to evolve and promote exercise physiology, scientifically, pedagogically and organisationally, are certainly reminiscent of the noble characters’ fight for justice in Alexandre Dumas's novel. I will try to refrain from digressing too much into the swashbuckler genre here, but Dumas's sentiment that ‘the merit of all things lies in their difficulty’ seems to resonate well with their scientific endeavours, as they repeatedly tackled scientific challenges that appeared insurmountable, either conceptually or methodologically (Asmussen, 1987; Åstrand, 1991). However, because a comprehensive account of their collective contributions would merit a whole monograph in its own right, I will only recapitulate one important aspect of their work from the formative years of Scandinavian exercise physiology here: the adaptative resetting of homeostasis across organ systems during exercise.

Erik Hohwü Christensen, universally known as ‘Hohwü’, was still an undergraduate student in natural history, chemistry, geography and geology when he joined Lindhard at the Gymnastics Theory Laboratory, shortly after the Rockefeller Institute had been inaugurated in 1928. Under Lindhard's supervision, he engaged in a series of studies that followed up on Krogh and Lindhard's work from the preceding decade that demonstrated extremely rapid cardiovascular and gas exchange kinetics following exercise onset. Initially, his efforts were focused on enhancing the functionality of Krogh's bicycle ergometer (Krogh, 1913) and refining Krogh and Lindhard's nitrous oxide rebreathing technique developed for the measurement of cardiac output (Krogh & Lindhard, 1912). While doing this, Hohwü ensured an unprecedented standardisation of experimental conditions, exercising stringent control over variables such as diet, meal timings and rest periods prior to measurements. As an alternative to the nitrous oxide rebreathing technique, Hohwü adapted the relatively new acetylene rebreathing method for measurements during exercise (Christensen, 1931*a*, 1932 *a*), and soon Hohwü expanded from mere methodologically based studies to mechanistic studies that focused not only on cardiac output, but also on blood glucose, respiration and thermoregulation (Christensen, 1931*b*, 1931 *c*, 1931 *d*, 1931 *e*, 1932 *b*). However, while Krogh and Lindhard's studies had mostly focused on exercise at moderate intensity in moderately fit men approaching middle age (themselves), Hohwü continued the longstanding Scandinavian tradition of self‐experimentation within physiology, to conduct studies at (near‐)maximal intensity in highly fit, young athletes, that is, himself and three fellow students that he knew from his dormitory, including Marius Nielsen. All these experiments were meticulously carried out using Krogh's now‐optimised bicycle ergometer, ensuring well‐defined workloads. Most critically, and unlike other contemporary studies that typically captured measurements during transitional states—such as at the onset of exercise, immediately following exertion or during short breaks—Hohwü’s work was characterised by extended continuous exercise sessions that facilitated the acquisition of steady state measurements, to compare and distinguish the physiological changes that take place during rest, exercise at well‐defined intensities, and recovery. Hohwü graduated in 1931, majoring in gymnastic theory, and defended his doctoral thesis based on the abovementioned studies later the same year. Marius Nielsen also graduated in 1931, majoring in natural history, and soon began conducting his experiments at the Gymnastics Theory Laboratory, focusing on the work of breathing during exercise (Nielsen, 1936*a*). Soon, an opportunity arose that would prove pivotal to their continued work.

Since World War I, there had been an extraordinary surge in enthusiasm for sports, fuelled by the belief in athleticism as a crucial factor for health. However, some critics questioned whether these efforts genuinely contributed to improving health, highlighting the lack of clear evidence on whether athletic sports had a positive or negative impact on the body. The ambiguity surrounding the physiological effects of strenuous exercise and the safe limits of such exertion prompted the League of Nations' Health Organization to convene a meeting in Copenhagen in January 1931. Here, it was acknowledged that the specific changes occurring in the body under extreme athletic strain remained largely unexplored. To address this, experts at the meeting recommended that the Health Organization initiate a comprehensive study into various physiological responses to maximum muscular effort. Krogh and Lindhard, who attended the meeting, proposed a 2‐year research programme to investigate these phenomena and subsequently received funding to continue Hohwü and Nielsen's research. The preliminary results were presented to the Health Organization in Geneva in 1934, encompassing a comprehensive 32‐page report (Christensen et al., 1934), which is also an early account of the oxygen transport cascade, highlighting the convective and diffusive steps for oxygen transport from ambient air to working muscle in relation to exercise performance.

The funding from the League of Nations' Health Organization enabled Krogh to hire both Hohwü and Nielsen as research assistants at the Zoophysiological Laboratory. Nielsen utilised this opportunity to also focus on the chemical regulation of ventilation (Nielsen, 1936*b*). These studies were not particularly focused on exercise as such, but exercise was used along with hypobaric hypoxia and experimentally induced metabolic acidosis (by orally administered ammonium chloride) as a means for uncovering the mechanisms regulating ventilation in humans. Specifically, Nielsen aimed to determine whether CO_2_ or H^+^ was the primary chemical stimulus that increased the ‘excitability’ of the respiratory centre to enhance breathing in these different conditions, a matter of great controversy during those years. During exercise, it was believed that either the increased CO_2_ or H^+^ production in the contracting skeletal muscle (and other working tissues) could increase $P_{\mathrm{aC}O_{2}}$ and/or reduce arterial pH, which would then increase ventilation. Lindhard and Krogh, in particular, had both placed their bets on $P_{\mathrm{aC}O_{2}}$, but much to Nielsen's surprise, he found that both $P_{\mathrm{aC}O_{2}}$ and arterial pH remained relatively constant during the prolonged strenuous exercise sessions to which he subjected Hohwü and his fellow students. When Nielsen presented this to Krogh, admitting that he did not understand how to grasp these findings, Krogh replied, ‘No, I don't understand that either! You must find out!’ Krogh said this in such a compelling manner that it remained a source of inspiration for Nielsen for much of his future work (Schmidt‐Nielsen, 1995).

Nielsen defended his doctoral thesis on the regulation of respiration in 1936. While he did not succeed in conclusively determining whether CO₂ or H⁺ was the primary stimulus governing the regulation of ventilation, much pointed towards CO_2_. However, the stimulus for the increased ventilation during any of the examined conditions was clearly not an increase in $P_{\mathrm{aC}O_{2}}$ per se, but rather seemed to involve an altered sensitivity of the respiratory centre to CO₂ (Nielsen, 1936*b*). Curiously, Nielsen followed up with a study on thermoregulation during exercise, to challenge a common viewpoint that the increase in core temperature during exercise represents a failure of thermoregulation (Nielsen, 1938). Hohwü’s previous study on core temperature during prolonged strenuous exercise, showing increases in temperature to levels that would be considered life‐threatening in a clinical setting, had not done much to resolve this (Christensen, 1931*c*). In a room that was carefully thermoregulated to maintain consistent air temperature and humidity, the young scientists cycled vigorously at various intensities on Krogh's bicycle ergometer, which was suspended on a scale for the continuous measurement of weight loss due to perspiration, while rectal and skin temperatures were continuously measured (Nielsen, 1936*a*, 1936 *b*). Rectal temperature increased proportionally with exercise intensity, independently of differences in the conditions for heat loss, while changes in skin temperature remained relatively minor (Nielsen, 1938). In fact, body temperature was kept within much narrower limits at a given exercise intensity than at rest. Nielsen could thus conclude that the rise in body temperature during exercise is a regulatory response and not the result of a failure in thermoregulation (Nielsen, 1938). Together, Nielsen's studies on ventilation and thermoregulation, conducted under carefully controlled conditions and at a well‐defined steady state of exercise as established by Hohwü, pointed towards highly regulated exercise adaptations.

While all this was going on, Asmussen, who was slightly younger than Hohwü and Nielsen, had started attending lectures at the Rockefeller Institute as a student of natural history. He became acquainted with Hohwü who knew that Lindhard needed an assistant to develop a method for isolating frog skeletal muscle fibres. Asmussen thus joined the Gymnastics Theory Laboratory as an unpaid assistant, and soon he was able to isolate muscle fibres from the frog's semitendinosus muscle, which then allowed him to conduct a series of studies on isometric and isotonic contractions. He continued spending his afternoons and evenings doing this after he majored in gymnastic theory in 1931 while earning his livelihood as a secondary school teacher of natural history and physical education, and in 1933 he defended his doctoral thesis on this work. By 1935, Asmussen had finally landed a position as a full‐time research assistant at the Gymnastics Theory Laboratory, this time with an actual salary, so that he could quit his job as a secondary school teacher and focus more wholly on his research. However, in the meantime, Lindhard had invited the renowned German neurophysiologist Fritz Buchtal (1907–2003) to join the laboratory, in part because of the increasing hostility towards the Jewish community in Germany. Buchtal's expertise naturally drove the focus at the laboratory more in the direction of electrophysiological studies of skeletal muscle, which prompted Asmussen to concentrate on the integrative physiology of the human body in response to exercise instead. Asmussen thus conducted a study on the cardiopulmonary response to static exercise by leg presses against an angle lever with considerable loads. The study showed that oxygen uptake, ventilation and cardiac output showed variable changes during exercise depending on the workload, but they all increased sharply immediately after, which was attributed to the mechanical constraint of blood flow by the actively engaged muscles (Asmussen & Hansen, 1938). The thematic overlap between Hohwü and Nielsen were obvious to anyone, and soon the three started working closely together. Thus, by 1936, the memorable trio had finally been formed!

Initially, Hohwü, Nielsen and Asmussen turned their attention towards matters deemed urgent at the time, in particular the mechanisms behind the increase in cardiac output during exercise. A prevalent theory suggested that vasodilatation in working muscle triggered a reduction in arterial blood pressure, which then led to an increase in cardiac output through baroreflex feedback. However, this could not explain the increase in stroke volume previously reported by Hohwü (Christensen, 1931*e*), and furthermore, the arterial baroreflex feedback loop had not been conclusively demonstrated in humans, neither during postural changes nor during steady state exercise. Moreover, the existing studies often uncritically assumed that heart rate was an accurate reflection of cardiac output, a notion also inconsistent with Hohwü’s earlier studies (Christensen, 1931*d*, 1931*e*). In the basement under the north wing of the Rockefeller Institute, the trio devised an experimental set‐up allowing for the manipulation of blood volume distribution through pneumatic thigh cuff inflation and deflation and by the use of a tilt table (enabling upright, supine and head‐down measurements), reconstructed from a resuscitation table previously designed by Krogh in one of his less successful scientific endeavours (Krogh, 1934). This set‐up also permitted the measurement of oxygen uptake, cardiac output (by Hohwü’s modified acetylene rebreathing technique), and blood volume changes through water displacement plethysmography. They mostly used exercise protocols that involved rhythmic arm extensions against an elastic resistance, or by employing Krogh's bicycle ergometer.

The routine was such that, after completing their teaching duties around 10 a.m., The Three Musketeers would commence the day's experiments, during which they took turns serving as experimental subjects. Following lunch, they dedicated time to analysing the data obtained. The set‐up and measurements were continually refined to achieve reproducible results; even so, one experimental day would generally yield only one measurement point for a given variable (Asmussen, 1987). Therefore, although their papers often report results from relatively few, in this case three subjects, these results are usually based on several months of daily experimentation. When it came to drafting manuscripts based on the the data, they would sit together at a desk in the late afternoons and evenings; Nielsen, having the neatest handwriting, would write with a pencil as they collectively formulated each sentence of the paper, while Hohwü, who came from a Danish‐minded family in Southern Jutland—a region that had not become part of Denmark until the Reunification in 1920—and had attended a German school, ensured that the paper's language, written in German, was impeccable (Asmussen, 1987). Krogh was a regular visitor, keen to showcase the experimental set‐up to scientists from around the world, and in the evenings, he would often stop by to discuss results and ideas, contributing to further optimisation of the experiments (Asmussen, 1987; Schmidt‐Nielsen, 1995).

In their collaborative experiments conducted from 1936 and approximately 5 years forth, The Three Musketeers demonstrated that thigh cuff deflation in the sitting position and a postural change from supine to upright both result in blood displacement to the lower extremities with a concomitant increase in heart rate, and vice versa when moving from upright to the supine posture (Asmussen et al., 1938, 1939a *a*). Despite a higher heart rate in the upright position, cardiac output was found to be lower due to a lower stroke volume than in the supine position, even though oxygen uptake was similar (Asmussen et al., 1939*b*). Additionally, superimposing arm exercise during postural changes while restricting venous return by thigh cuff inflation, caused heart rate to vary with posture in relation to differences in stroke volume, but it still increased with exercise in all postures (Asmussen & Christensen, 1939; Asmussen et al., 1939*c*). The baroreflex feedback loop could thus be effectively demonstrated in humans, and it seemed to remain equally effective for regulating heart rate during exercise compared to rest. Hence, as Nielsen had demonstrated for ventilation and thermoregulation, the changes in cardiovascular function during exercise seemed to reflect highly regulated phenomena.

In another study, about half of the working musculature was isolated from the rest of the circulation by thigh cuff inflation during exercise on Krogh's bicycle ergometer. Despite a significant reduction in oxygen uptake, cardiac output and heart rate were barely affected (Asmussen et al., 1940). This indicated that the determinant of the cardiac output increase during exercise was the workload. In a subsequent study, a similar increase in cardiac output was observed, both in the healthy state and in the case of impaired proprioceptive feedback due to posterior fascicle demyelination (neurosyphilis) when muscle contractions were induced by electrical stimulation (Asmussen et al., 1943*a*). Together these studies showed that during steady state light‐to‐moderate exercise, it was not vasodilatation in the working muscle nor cortical feed‐forward impulses that accounted for the increase in cardiac output. Given that no substances produced in the muscles below the inflated cuffs could enter the main circulation while the nervous connections remained intact, a neural feedback mechanism related to the volume of involved musculature, but unrelated to proprioception, seemed to be at play. Following up on the studies from Nielsen's doctoral thesis, similar observations were made regarding the ventilatory response to exercise. Thus, during light and moderate exercise on  Krogh's bicycle ergometer, the reduction in oxygen uptake evoked by thigh cuff inflation did not affect the ventilatory response to exercise (Asmussen, Hohwü Christensen et al., 1943). Furthermore, a similar ventilatory response was observed during electrically induced muscle contractions, both in the healthy state and in the case of posterior fascicle demyelination (Asmussen et al., 1943*b*). Hence, the steady state ventilatory response to exercise also seemed to be triggered by a non‐proprioceptive neural feedback mechanism. By the early 1940s, The Three Musketeers had thus demonstrated that ventilation, thermoregulation and cardiovascular function remain tightly regulated during exercise, involving feedback mechanisms originating within the working skeletal muscle that function to reset this regulation according to the given workload. However, they could not identify the exact stimulus that triggered this feedback mechanism.

In 1941, that is, at the peak of The Three Musketeers’ collaborative studies, Hohwü decided to depart to Sweden to pursue a professorship at the Royal Gymnastic Central Institute (GCI) in Stockholm, thus marking the end of this formative period of Scandinavian exercise physiology. Apart from a brief leave of absence from GCI in the early 1960s, Hohwü remained there until his retirement in 1970. However, to quote Dumas, ‘Friendship is a sacred bond that cannot be broken by distance or time’, and the trio remained in touch. Notably, Asmussen and Nielsen, who both became professors at the Gymnastics Theory Laboratory in 1961 and 1965, respectively, continued to investigate the elusive stimulus within working muscle that seemingly functioned to reset homeostatic regulation, initially naming it the ‘work stimulus’ (Asmussen & Nielsen, 1958), but later using the term ‘work factor’ (Asmussen, 1965), thus aligning with the contemporary terminology in exercise endocrinology (Goldstein, 1965; Ingle, 1955). Later, Hohwü’s students, notably Bengt Saltin (1935–2014), followed up on another series of Hohwü’s early studies on skeletal muscle fuel utilization during exercise, thereby integrating glucose homeostasis into this paradigm. However, that is a different story for another time.

When reflecting on the collaboration between The Three Musketeers within the context of Alexandre Dumas's 1842 novel, I have often wondered: who is who? One might liken Hohwü to Athos, the noble and naturally leading figure. Nielsen could be compared to Aramis, the quieter and more thoughtful innovator. As for Asmussen, rather than resembling Porthos, the jovial and boisterous member, he might be better represented as D'Artagnan. Hence, during these formative years, the younger Asmussen grew to become Hohwü and Nielsen's equal, and subsequently a driving force for their collaboration that served as the group's visionary (Saltin, 1991). This is well‐exemplified by an opening talk of his at a symposium entitled ‘Physiology of Muscular Exercise’ held in Dallas in 1966, in which he compiled the results of The Three Musketeers to elegantly formulate the concept of adaptive resetting of homeostasis during exercise (Asmussen, 1967): ‘For every state of physical exercise, there is a carefully controlled level of pulmonary function, ventilation, cardiac output and deep body temperature. These levels are maintained at least as precisely as the resting level, and the controlling feedback systems are the same in exercise as during rest; only the set‐point has been changed.’

At the present day, it is evident that this adaptive resetting of homeostasis during exercise should be considered a universal phenomenon involving myriad neural and non‐neural mechanisms, encompassed by the evolving concept of interorgan crosstalk (Hargreaves, 2021; Severinsen & Pedersen, 2020). Returning to the questions posed at The League of Nations meeting in 1931, it is thus clear that exercise is not merely a disturbance that organ systems must counteract, eliciting abnormal or extreme responses. Rather, every organ of the healthy body is ‘designed’ to accommodate to the steady state of exercise, both in its own right and through its interaction with other organs—‘one for all, and all for one!’

## AUTHOR CONTRIBUTIONS

Sole author.

## CONFLICT OF INTEREST

The author has no conflict of interest to declare.

## FUNDING INFORMATION

None.

## References

[eph13558-bib-0001] Asmussen, E. (1965). Muscular exercise. In W. O. Fenn , & H. Rahn (Eds.), Handbook of physiology. Section 3: Respiration. Volume II (pp. 939–978). American Physiological Society.

[eph13558-bib-0002] Asmussen, E. (1967). Exercise: General statement of unresolved problems. Circulation Research, 20–21, I2–I6.

[eph13558-bib-0003] Asmussen, E. (1987). Gymnastikstudiet og det gymnastikteoretiske laboratorium ved Københavns Universitet. Tilbageblik på trekvart århundredes hændelser. Idrættens Forskningsråd.

[eph13558-bib-0004] Asmussen, E. , & Christensen, E. H. (1939). Einfluß der Blutverteilung auf den Kreislauf bei körperlicher Arbei. Skandinavisches Archiv Für Physiologie, 82, 185–192.

[eph13558-bib-0005] Asmussen, E. , Christensen, E. H. , & Nielsen, M. (1938). Über die Beeinflussung der Pulsfrequenz durch Änderungen des arteriellen Blutdruckes. Skandinavisches Archiv Für Physiologie, 79, 32–38.

[eph13558-bib-0006] Asmussen, E. , Christensen, E. H. , & Nielsen, M. (1939a). I. Pulsfrequenz und Körperstellung. Skandinavisches Archiv Für Physiologie, 81, 190–203.

[eph13558-bib-0007] Asmussen, E. , Christensen, E. H. , & Nielsen, M. (1939b). III. Über die Kreislaufinsuffizienz in stehender Stellung bei normalem arteriellen Druck und herabgesetztem Minutenvolumen. Skandinavisches Archiv Für Physiologie, 81, 214–224.

[eph13558-bib-0008] Asmussen, E. , Christensen, E. H. , & Nielsen, M. (1939c). IV. Die Bedeutung der Körperstellung für die Pulsfrequenz bei Arbeit. Skandinavisches Archiv Für Physiologie, 81, 225–233.

[eph13558-bib-0009] Asmussen, E. , Christensen, E. H. , & Nielsen, M. (1940). Kreislaufgröße und cortikal‐motorische Innervation. Skandinavisches Archiv Für Physiologie, 83, 181–187.

[eph13558-bib-0010] Asmussen, E. , & Hansen, E. (1938). Über den Einfluß statischer Muskelarbeit auf Atmung und Kreislauf. Skandinavisches Archiv Für Physiologie, 78(1), 283–303.

[eph13558-bib-0011] Asmussen, E. , Hohwü Christensen, E. , & Nielsen, M. (1943). Humoral or nervous control of respiration during muscular work? Acta Physiologica Scandinavica, 6(2–3), 160–167.

[eph13558-bib-0012] Asmussen, E. , & Nielsen, M. (1958). Pulmonary ventilation and effect of oxygen breathing in heavy exercise. Acta Physiologica Scandinavica, 43(3–4), 365–378. 13582656 10.1111/j.1748-1716.1958.tb01601.x

[eph13558-bib-0013] Asmussen, E. , Nielsen, M. , & Wieth‐Federsen, G. (1943a). On the regulation of circulation during muscular work. Acta Physiologica Scandinavica, 6(4), 353–358.

[eph13558-bib-0014] Asmussen, E. , Nielsen, M. , & Wieth‐Pedersen, G. (1943b). Cortical or reflex control of respiration during muscular work? Acta Physiologica Scandinavica, 6(2–3), 168–175.

[eph13558-bib-0015] Åstrand, P. O. (1991). Influence of Scandinavian scientists in exercise physiology. Scandinavian Journal of Medicine & Science in Sports, 1(1), 3–9.

[eph13558-bib-0016] Christensen, E. H. (1931a). Beiträge zur Physiologie schwerer körperlicher Arbeit: III. Mitteilung: Gasanalytische Methoden zur Bestimmung des Herzminutenvolumens in Ruhe und während körperlicher Arbeit. Arbeitsphysiologie, 4, 175–202.

[eph13558-bib-0017] Christensen, E. H. (1931b). Beiträge zur Physiologie schwerer körperlicher Arbeit: I. Mitteilung: Der Blutzucker während und nach körperlicher Arbeit. Arbeitsphysiologie, 4, 128–153.

[eph13558-bib-0018] Christensen, E. H. (1931c). Beiträge zur Physiologie schwerer körperlicher Arbeit: II. Mitteilung: Die Körpertemperatur während und unmittelbar nach schwerer körperlicher Arbeit. Arbeitsphysiologie, 4, 154–174.

[eph13558-bib-0019] Christensen, E. H. (1931d). Beiträge zur Physiologie schwerer körperlicher Arbeit: IV. Mitteilung: Die Pulsfrequenz während und unmittelbar nach schwerer körperlicher Arbeit. Arbeitsphysiologie, 4, 453–469.

[eph13558-bib-0020] Christensen, E. H. (1931e). Beiträge zur Physiologie schwerer körperlicher Arbeit: V. Mitteilung: Minutenvolumen und Schlagvolumen des Herzens während schwerer körperlicher Arbeit. Arbeitsphysiologie, 4, 470–502.

[eph13558-bib-0021] Christensen, E. H. (1932a). Beiträge zur Physiologie schwerer körperlicher Arbeit: VII. Mitteilung: Über die Brauchbarkeit der Acetylenmethode zur Bestimmung des Herzminutenvolumens während körperlicher Arbeit. Arbeitsphysiologie, 5, 479–488.

[eph13558-bib-0022] Christensen, E. H. (1932b). Beiträge zur Physiologie schwerer körperlicher Arbeit: VI. Mitteilung: Der Stoffwechsel und die respiratorischen Funktionen bei schwerer körperlicher Arbeit. Arbeitsphysiologie, 5, 463–478.

[eph13558-bib-0023] Christensen, E. H. , Krogh, A. , & Lindhard, J. (1934). Recherches sur l'effort musculaire intense. Bull Trimest l'Organisation d'Hygiène la Société des Nations, 3, 407–439.

[eph13558-bib-0024] Goldstein, M. S. (1965). Muscular exercise and subsequent glucose utilization. In B. S. Leibel , & G. A. Wrenshall (Eds.) Nature and treatment of diabetes mellitus (pp. 308–321). Excerpta Medica Foundation.

[eph13558-bib-0025] Hargreaves, M. (2021). Exercise and health: Historical perspectives and new insights. Journal of Applied Physiology, 131(2), 575–588. 34166112 10.1152/japplphysiol.00242.2021

[eph13558-bib-0026] Ingle, D. J. (1955). Effect of endocrine glands on normal muscle work. American Journal of Physiology, 19(5), 724–728. 10.1016/s0002-9343(55)80015-0 13268471

[eph13558-bib-0027] Krogh, A. (1913). A bicycle ergometer and respiration apparatus for the experimental study of muscular work. Skandinavisches Archiv Für Physiologie, 30(3), 375–394.

[eph13558-bib-0028] Krogh, A. (1934). En vippebaare til kunstigt aandedræt. Militaerlaegen, 40, 107.

[eph13558-bib-0029] Krogh, A. , & Lindhard, J. (1912). Measurements of the blood flow through the lungs of man. Skandinavisches Archiv Für Physiologie, 27(2), 100–125.

[eph13558-bib-0030] Nielsen, M. (1936a). Die Respirationsarbeit bei Körperruhe und bei Muskelarbeit. Skandinavisches Archiv Für Physiologie, 74, 299–316.

[eph13558-bib-0031] Nielsen, M. (1936b). Untersuchungen über die Atemregulation beim Menschen: Besonders mit Hinblick auf die Art des chemischen Reizes. Skandinavisches Archiv Für Physiologie, 74, 87–208.

[eph13558-bib-0032] Nielsen, M. (1938). Die Regulation der Körpertemperatur bei Muskelarbeit. Skandinavisches Archiv Für Physiologie, 79(2), 193–230.

[eph13558-bib-0033] Saltin, B. (1991). Erling Asmussen. 6 August 1907 –12 April 1991. In Det Kongelige Danske Videnskabernes Selskab, Oversigt over Selskabets Virksomhed, 1991–1992 (pp. 139–144) *Annual Report with an English Summary*. Munksgaard.

[eph13558-bib-0034] Schmidt‐Nielsen, B. (1995). August and Marie Krogh: Lives in science. Springer.

[eph13558-bib-0035] Severinsen, M. C. K. , & Pedersen, B. K. (2020). Muscle‐organ crosstalk: The emerging roles of myokines. Endocrine Reviews, 41(4), 594–609. 32393961 10.1210/endrev/bnaa016 PMC7288608

